# *Plinia cauliflora* Leaf Extract Promotes Wound Healing Through Anti‐Inflammatory, Antioxidant, and Antibacterial Actions

**DOI:** 10.1002/cbdv.202501294

**Published:** 2025-08-30

**Authors:** Priscila de Lima Paula, Mariana Hauck Vianna, Júlia Bertolini Fajardo, Ana Barbara Polo, Lara Melo Campos, Thayná Gomes Ferreira, Ari Sérgio de Oliveira Lemos, Thalita de Freitas Souza, Lívia Rodrigues Gamarano, Maria Clara Machado Resende Guedes, Samira Aparecida Coelho Souza, Ayrton Senna Pinheiro, Leandro De Santis Ferreira, José Maria Barbosa‐Filho, Elaine Soares Coimbra, Gilson Costa Macedo, Luciana Moreira Chedier, Rodrigo Luiz Fabri

**Affiliations:** ^1^ Laboratory of Bioactive Natural Products Department of Biochemistry Institute of Biological Sciences Federal University of Juiz de Fora Juiz de Fora Minas Gerais Brazil; ^2^ Department of Parasitology, Microbiology, and Immunology Institute of Biological Sciences Federal University of Juiz de Fora Juiz de Fora Minas Gerais Brazil; ^3^ Department of Botany Institute of Biological Sciences Federal University of Juiz de Fora Juiz de Fora Minas Gerais Brazil; ^4^ Laboratory of Quality Control of Medicines Department of Pharmacy Federal University of Rio Grande Do Norte Natal Rio Grande do Norte Brazil; ^5^ Research Institute for Drugs and Medicines Federal University of Paraíba, Campus I, Castelo Branco João Pessoa Paraíba Brazil

**Keywords:** medicinal plants, phenolic compounds, *Plinia cauliflora*, skin wounds, wound healing

## Abstract

*Plinia cauliflora* (DC.) Kausel (jabuticaba) is traditionally used by Brazilian indigenous communities for therapeutic purposes, including the treatment of wounds and inflammation. This study investigated the phytochemical composition and biological activities of an ethanolic extract derived from *P. cauliflora* leaves (ethanolic extract from *P. cauliflora* leaves [EEPC]). UHPLC‐MS phytochemical analysis revealed 13 active phenolic compounds, with gallic acid being the most prevalent at 319.5 µg/mg extract. EEPC demonstrated significant antioxidant activity (IC_50_ = 4.42 ± 0.91 µg/mL, 2,2‐diphenyl‐1‐picrylhydrazyl (DPPH) assay), reducing reactive oxygen species (ROS) generation by 63% and inhibiting lipid peroxidation by 63.69%. Furthermore, it exhibited anti‐inflammatory effects by decreasing IL‐1β and TNF‐α levels by 40% and 74%, respectively. Antimicrobial activity was observed against *Pseudomonas aeruginosa* and *Candida albicans* with a minimum inhibitory concentration (MIC) of 250 µg/mL. EEPC also reduced *Staphylococcus aureus* biofilm adhesion by 95.40%. Notably, EEPC enhanced fibroblast migration by 95.40%, suggesting its potential for wound healing. These results highlight the therapeutic potential of EEPC and the value of Brazilian biodiversity for biomedical applications.

## Graphical abstract

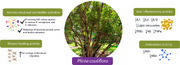

## 1 Introduction

The skin, the human body's largest organ, acts as a vital and intricate physical barrier, shielding the body from external dangers. Its protective function is paramount, making rapid integrity restoration crucial following injury [1]. Physiologically, healing progresses through distinct phases: inflammatory, proliferative, and maturation. Essentially, tissue remodeling involves a robust inflammatory response in the affected area, subsequently stimulating cells crucial for extracellular deposition and wound closure [2].

Skin wounds in general are one of the major problems affecting the population. They can be caused by a variety of factors, such as burns or traumatic injuries, and require significant investment from the public health system for proper management [3]. These conditions not only affect an individual's productivity but also their social interaction, autonomy, and self‐esteem [4].

Conventional wound management continues to rely heavily on the utilization of non‐steroidal anti‐inflammatory drugs (NSAIDs) or systemic antibiotics, which are employed to mitigate inflammation and infection. However, these pharmaceutical interventions have been associated with well‐documented adverse effects, including the development of microbial resistance, hypersensitivity reactions, and contact dermatitis [5]. Building on these shortcomings, several recent ethnopharmacological surveys emphasize that Latin‐American medicinal plants, especially members of the Myrtaceae family such as *Plinia cauliflora*, are traditionally applied to skin injuries because they combine antimicrobial and inflammation‐modulating activities that act at multiple stages of tissue repair [6].

Preparations made from jaboticaba, including fruits, peels, and leaves, have been shown to accelerate re‐epithelialization. This effect is supported by ethnobotanical fieldwork and synthetic reviews published in 2024. These preparations are rich in antioxidant anthocyanins and ellagitannins, which are believed to stabilize the extracellular matrix (ECM) and regulate cytokine balance [7].

Despite the recognized bioactivity of such species, their commercial utilization remains limited. A significant proportion of native flora is characterized by a high degree of perishability, with harvesting occurring on a yearly basis [8]. Additionally, non‐edible components, such as leaves and branches, are usually discarded. The focus of research on these underutilized plant parts can be considered a sustainable strategy for the purpose of adding value to agro‐industrial by‐products and reducing waste [9].

This approach is in alignment with the United Nations Sustainable Development Goal 12 (Responsible Consumption and Production), which emphasizes the reduction of resource use, the minimization of waste generation, and the promotion of sustainable practices across the supply chain. A study of the leaves of *P. cauliflora*, a species native to the Brazilian Atlantic Forest, serves to reinforce the concept that the conservation of biodiversity is contingent upon scientific knowledge and its subsequent valorization. Consequently, phytochemical and pharmacological investigations not only promote innovation but also contribute to achieving the 2030 Agenda for Sustainable Development [10].

*P. cauliflora* (DC.) Kausel, commonly known as jabuticaba, is a native plant of the Brazilian Atlantic Rainforest. Traditionally, it is used in indigenous medicine to treat inflammation, asthma, diarrhea, tonsillitis, female genitourinary disorders, and to promote wound healing [11, 12]. The fruit is highly valued, and the leaves are rich in phenolic compounds, such as anthocyanins, ellagic acid, gallic acid, ellagitannins, and flavonoids [13]. These compounds contribute to the plant's notable antioxidant, anti‐inflammatory, and antimicrobial properties [14, 15, 16]. Despite these characteristics, most research on *P. cauliflora* has concentrated on the biological effects of its fruit [6].

Studies on the ethanolic extract of *P. cauliflora* leaves (EEPC), which is rich in flavonoids, show that it is an excellent source of antioxidant compounds that can be used in formulations to prevent skin ageing, protect against sun damage, and promote wound healing [17]. Although the antioxidant and anti‐inflammatory properties of *P. cauliflora*, as well as its effect on bacteria responsible for wound infection and cell migration, are safe, these effects need to be further investigated.

On the basis of reports in the literature, the extract of *P. cauliflora* leaves is believed to exhibit potent antimicrobial, antioxidant, anti‐inflammatory, and healing properties, which are attributed to its high phenolic content. This study aimed to explore the extract's potential for treating skin wounds. UPLC‐QTOF‐MS and HPLC‐diode array detector (DAD) techniques were employed to identify and quantify its major constituents. Furthermore, the extract's antimicrobial, antioxidant, anti‐inflammatory, and healing properties were evaluated with a focus on topical application in wound care.

## 2 Results and Discussion

### 2.1 Total Phenolic, Tannin, and Flavonoid Content

On the basis of the known composition of this species, *P. cauliflora* leaves are recognized for their high content of phenolic compounds, especially tannins and flavonoids [13, 18]. Examples of these classes of compounds often included quercetin and rutin, which are known for their notable antioxidant, anti‐inflammatory, and antimicrobial effects [19, 20].

Total phenolics, tannins, and flavonoids were measured in EEPC. About total phenolics, 310.42 ± 8.75 µg/mg extract in tannic acid equivalents (TAEs) were found in EEPC. The tannin assay showed 108.56 ± 0.39 µg/mg extract in TAEs, and the flavonoid assay showed 36.80 ± 3.44 µg/mg extract in rutin equivalents.

A study identified a phenolic content exceeding 45% in *P. cauliflora* leaf extract, alongside gallic and ellagic acids, accounting for 509 mg/g of total tannins in the leaf [19]. Aglycones and glycosylated flavonoids, such as quercetin and myricetin, respectively, and hydrolysable tannins were also identified in another study [21].

### 2.2 Phytochemical Profile

UPLC‐QTOF‐MS analysis identified 13 substances, detailed in Table 1, with peaks numerically labeled by retention time. These include quinic acid (1), epicatechin (2), casuarictin (3), myricetin derivatives (4 and 5), myricitrin (6), quercetin derivatives (7‐9), quercitrin (10), rutin (11), quercetin (12), and gallic acid (13). This finding is consistent with the results of earlier studies that reported a high concentration of phenolic compounds in *P. cauliflora* leaves [22, 23, 24].

**TABLE 1:** Compounds identified in the ethanolic extract of leaves from *Plinia cauliflora* (EEPC) by UHPLC‐QTOF‐MS.

N	Compound	RT^*^ (min)	Mode	Mass fragmentation	Mass error^**^ (ppm)	References
1	Quinic acid	2.743	[M + H]^+^	193.0738 (M + H)	14.06	Paula et al. [23]
2	Epicatechin	12.604	[M + H]^+^	291.0910 (M + H)	14.47	Paula et al. [23]
3	Casuarictin	12.700	[M + H]^+^	954.1292 (M + NH_4_)	2.99	Paula et al. [23]
4	Myricetin‐3‐*O*‐hexoside	14.390	[M + H]^+^	481.1032 (M + H)	10.62	Paula et al. [23]
5	Myricetin‐3‐*O*‐pentoside	15.036	[M + H]^+^	451.0930 (M + H)	8.89	Paula et al. [23]
6	Myricitrin	15.234	[M + H]^+^	465.1091 (M + H); 319.0494 (M + H‐rhamnose)	12.70	Paula et al. [23]
7	Quercetin‐3‐*O*‐Hexoside	15.301	[M + H]^+^	465.1084 (M + H); 303.0359 (M + H‐hexose)	11.2	Boso et al. [24]
8	Quercetin‐3‐*O*‐pentoside	15.798	[M + H]^+^	435.0970 (M + H); 303.0536 (M + H)	9.90	Paula et al. [23]
9	Quercetin‐3‐*O*‐pentoside isomer	15.980	[M + H]^+^	435.0979 (M + H); 303.0538 (M + H)	12.00	Paula et al. [23]
10	Quercitrin	16.295	[M + H]^+^	449.1136 (M + H); 303.0541 (M + H‐Rhamnose)	11.80	Paula et al. [23]
11	Rutin	17.637	[M + H]^+^	611.1458 (M + H)	−25.07	Paula et al. [23]
12	Quercetin	19.098	[M + H]^+^	303.0547 (M + H)	14.23	Galvão et al. [22]
13	Gallic acid	22.643	[M + H]^+^	209.1204	3.52	Paula et al. [23]

*Note*:*Retention time (Rt) in minutes.

** Mass error (ppm): The difference between the experimentally measured mass‐to‐charge ratio (*m*/*z*) and the theoretical exact mass, expressed in parts per million (ppm). This value indicates the accuracy of the mass measurement in high‐resolution mass spectrometry.

HPLC‐DAD methods were employed to identify and quantify gallic acid, epicatechin, rutin, and quercetin in EEPC. These compounds were identified by comparing their retention times with standards at 254 nm. Gallic acid was found to be the most concentrated phenolic compound (319.5 µg/mg extract). Rutin (74.0 µg/mg extract), epicatechin (17.55 µg/mg extract), and quercetin (1.19 µg/mg extract) were present in lower concentrations. These quantified phenolic compounds can serve as chemical markers for EEPC.

### 2.3 Cell Viability

Some cells are directly involved in the skin repair process, including macrophages and fibroblasts [25]. Considering the relevance of these cells in wound healing, we analyzed in vitro cell viability of EEPC using the MTT reduction assay. This assay determines cell viability by measuring the percentage of metabolically active cells capable of reducing MTT to formazan [26].

**FIGURE 1:**
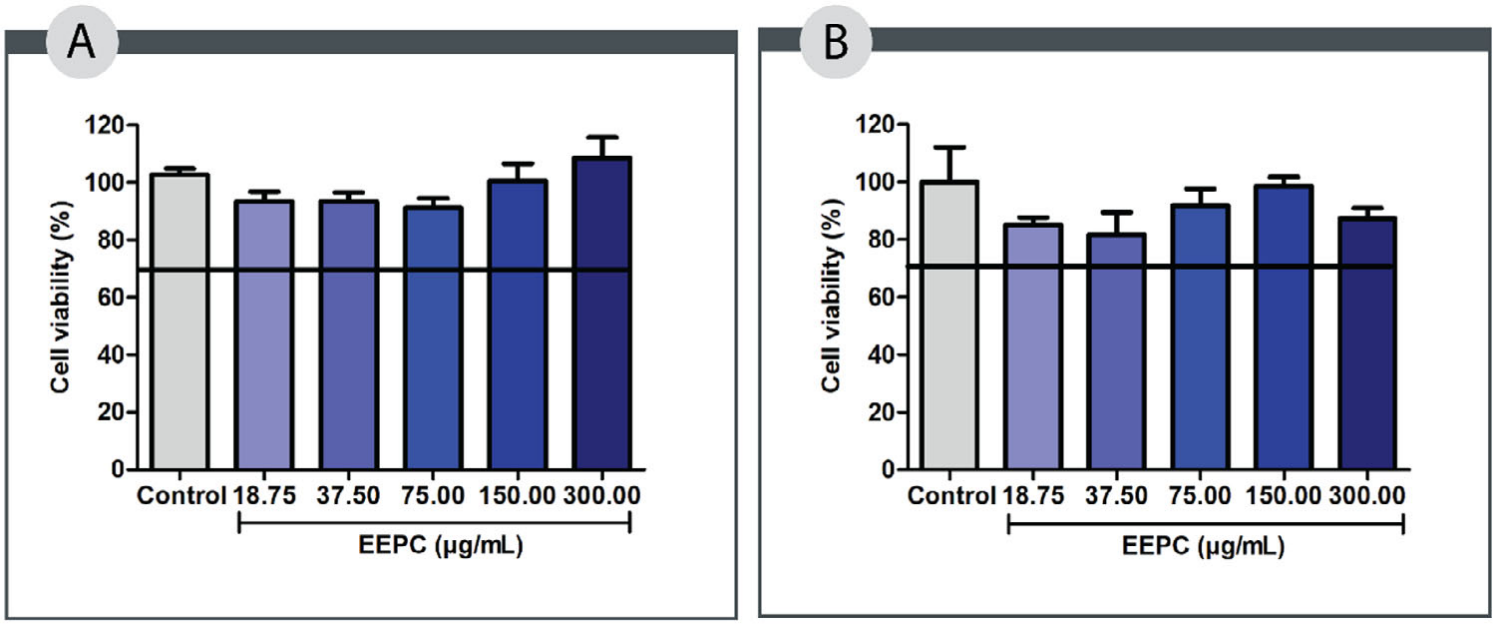
Evaluation of the cell viability in the peritoneal macrophages of the BALB/c (A) and fibroblasts L929 (B) after treatment with the ethanolic extract from *Plinia cauliflora* leaves (EEPC). Control—cells cultured in a medium added to the vehicle (DMSO). ANOVA followed by the Bonferroni test. The experiment was conducted in triplicate, and results are expressed as mean ± standard deviation (SD). Error bars represent SD.

According to international standards [27], an acceptable level of cell viability must exceed 70% of the cultured cells. As shown in Figure 1, all tested concentrations of EEPC (18.75–300.00 µg/mL) maintained cell viability above this threshold across the evaluated cell lines, suggesting a favorable safety profile. These findings support the potential use of EEPC in subsequent experimental studies. Consistent with our results, Pitz et al. demonstrated that a hydroalcoholic extract of jabuticaba peels promoted L929 fibroblast proliferation at concentrations of 25, 50, and 100 µg/mL after 48 h of exposure [12].

### 2.4 Antioxidant Properties

Long‐term production of reactive oxygen species (ROS) and nitrogen species can cause cellular damage and interfere with proper tissue repair. The release of pro‐inflammatory cytokines is mediated by the overproduction of ROS. Furthermore, it can affect fibroblast and keratinocyte homeostasis, reduce ECM deposition, and activate pro‐apoptotic proteins, resulting in cell death. The process of wound healing may, therefore, be greatly improved by substances that reduce the overproduction of free radicals [28, 29].

**FIGURE 2:**
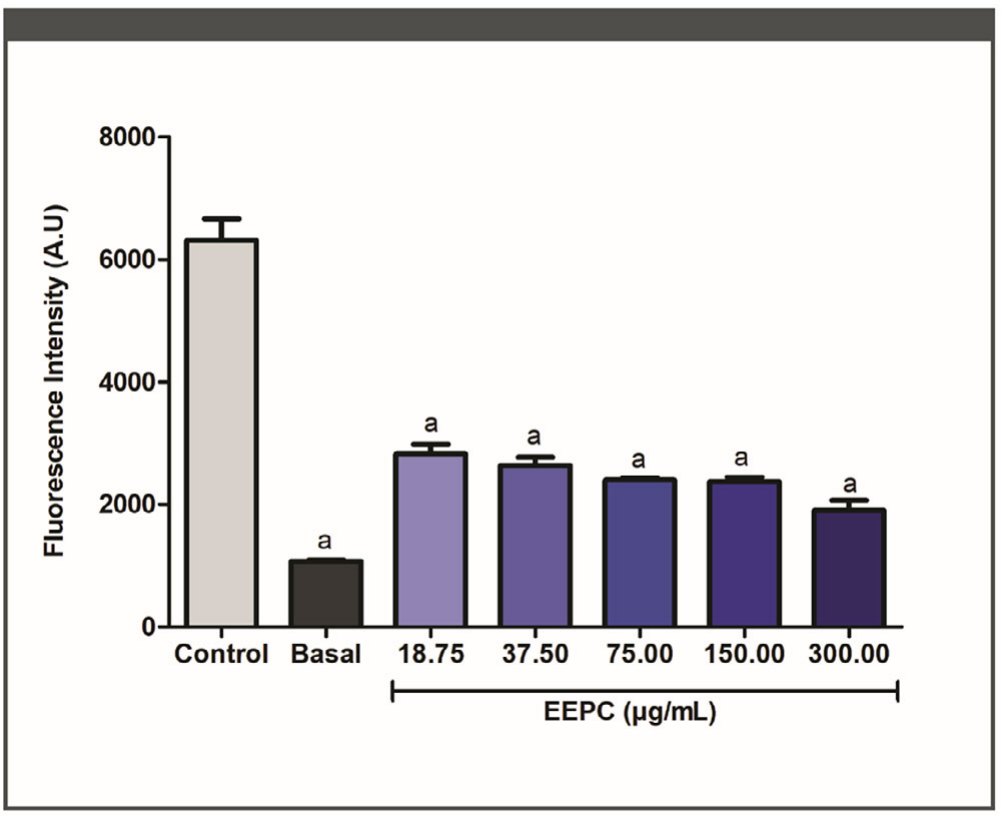
Evaluation of antioxidant activity of the ethanolic extract from *Plinia cauliflora* leaves (EEPC) through reduction of reactive oxygen species (ROS) levels in macrophages after treatment with the EEPC. Basal—Unstimulated cells treated with vehicle (DMSO). Control—Stimulated cells (IFN‐γ + LPS) treated with vehicle (DMSO). a—Statistical difference from control (*p* < 0.05). ANOVA followed by the Bonferroni test. The experiment was conducted in triplicate, and results are expressed as mean ± standard deviation (SD). Error bars represent SD.

Fluorometric tests were used to assess the effect of EEPC treatment on macrophage ROS generation. At each concentration tested, EEPC significantly decreased ROS production (Figure 2) (*p* < 0.05). EEPC reduced ROS generation by 63%, 56%, 53%, 51%, and 48% at 300.00, 150.00, 75.00, 37.50, and 18.75 µg/mL, respectively. These results are statistically equivalent to the baseline control (*p* < 0.05). The IC_50_ value obtained for the EEPC was 44.78 ± 1.38 µg/mL.

The radical scavenging activity of EEPC was evaluated by the 2,2‐diphenyl‐1‐picrylhydrazyl (DPPH) assay [30]. As shown in Table 2, the IC_50_ value of EEPC (4.42 ± 0.91 µg/mL) was indicative of a promising outcome. Although the results were statistically different from the positive controls (quercetin: 0.33 ± 0.12 µg/mL; rutin: 1.71 ± 0.22 µg/mL), there are reports in the literature on the DPPH radical scavenging activity of *P. cauliflora* that support the results described in this article. Souza‐Moreira et al. reported an antioxidant activity of approximately 90% for the ethanolic leaf extract of *P. cauliflora*, using the same method (DPPH radical) [19]. Other studies have also obtained promising antioxidant results when evaluating extracts from branches, bark, and fruits of the same species [31, 32]. The substantial antioxidant potential exhibited in the DPPH test indicates an elevated capacity for scavenging free radicals, a crucial factor in a healing environment. Excessive production of free radicals has been demonstrated to result in increased tissue damage, elevated inflammation risk, and impaired healing [33].

**TABLE 2:** Evaluation of the antioxidant potential of the ethanolic extract of *Plinia cauliflora* leaves (EEPC) through the 2,2‐diphenyl‐1‐picrylhydrazyl (DPPH) free radical scavenging, phosphomolybdenum complex reduction, and β‐carotene/linoleic acid system inhibition methods.

Samples	DPPH•—IC_50_ (µg/mL)	Phosphomolybdenum complex reduction			β‐Carotene/linoleic acid (38.46 µg/mL)		
		% Relative of rutin	% Relative of quercetin	% Relative of ascorbic acid	Inibition (%)	*F*1	*F*2
EEPC	4.42 ± 0.91^a^,^b^	199.72 ± 2.34	122.05 ± 1.43	41.44 ± 0.48	52.090 ± 7.380^a^	0.552 ± 0.370	0.618 ± 0.10
Rutin	1.71 ± 0.02	—	—	—	64.430 ± 2.400	0.570 ± 0.070	0.699 ± 0.11
Quercetin	0.33 ± 0.12	—	—	—	25.200 ± 0.900	0.590 ± 0.009	1.630 ± 0.32

*Note*: “a” is the statistical difference from positive control rutin (*p* < 0.05). “b” is the statistical difference from positive control quercetin (*p* < 0.05). ANOVA followed by the Bonferroni test. The experiment was conducted in triplicate, and results are expressed as mean ± standard deviation (SD).

The phosphomolybdenum complex reduction test, which indicates the antioxidant potential of phytoconstituents that are soluble or not in aqueous media, was also used to assess the total antioxidant capacity of EEPC [34]. For EEPC, the RRA% of quercetin and rutin were 122.05% ± 1.43% and 199.72% ± 2.34%, respectively. These findings indicate that the phytoconstituents of EEPC have antioxidant properties comparable to those of the positive controls.

**FIGURE 3:**
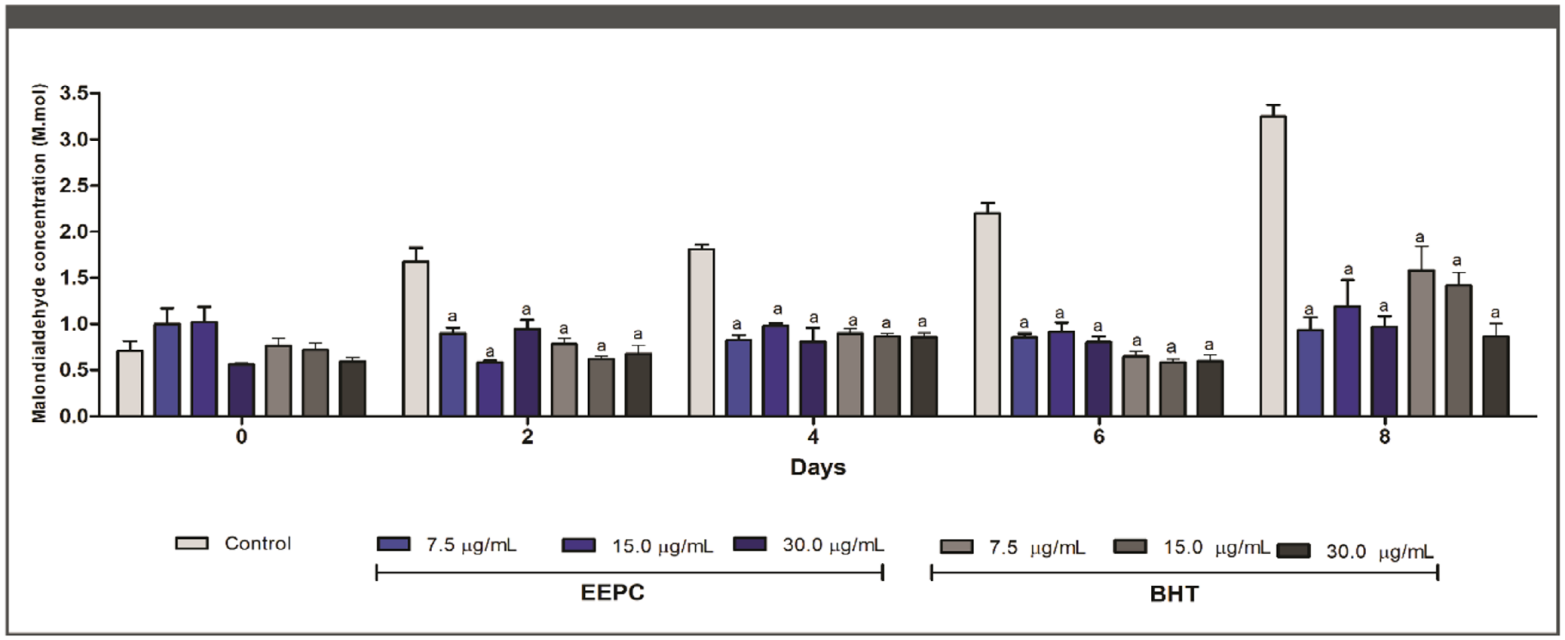
Inhibition of malondialdehyde formation in the presence of the ethanolic extract from *Plinia cauliflora* leaves (EEPC). Butylated hydroxytoluene (BHT) was employed as positive control. Control—treatment with vehicle. a—Statistical difference from vehicle (*p* < 0.05). ANOVA followed by the Bonferroni test. The experiment was conducted in triplicate, and results are expressed as mean ± standard deviation (SD). Error bars represent SD.

Aldehydes, compounds that are extremely harmful to cellular metabolism, are formed as a result of lipid peroxidation caused by an excess of free radicals [35]. Antioxidant samples are thought to prevent the formation of malondialdehyde (MDA), a biomarker of lipid peroxidation [36]. As shown in Figure 3, the ability of EEPC to reduce MDA formation was statistically higher than the negative control from Day 2. On Day 8, 30.00, 15.00, and 7.50 µg/mL EEPC reduced MDA formation by 57%, 52%, and 58%, respectively. These results were statistically similar to the corresponding doses of Butylated hydroxytoluene (BHT) used as a positive control. The β‐carotene/linoleic acid assay was also performed to evaluate the inhibition of lipid peroxidation by EEPC. When linoleic acid is oxidized, it forms radical structures that attack the double bonds of β‐carotene, resulting in a color change. However, when antioxidant compounds are added to the system, peroxidation of linoleic acid does not occur, and consequently the color of β‐carotene remains unchanged [37].

EEPC exhibited a peroxidation inhibition of 52.09% (Table 2), significantly higher compared to quercetin (25.20%—*p* < 0.05) and similar compared to rutin (64.43%—*p* > 0.05). The parameters *F*1 and *F*2 were also employed to evaluate antioxidant activity, with their values ideally approaching 0 and remaining below 1 to obtain an acceptable antioxidant activity [38]. According to the *F*1 parameter, the antioxidant activity of the extract was statistically similar when compared to rutin and quercetin (*p* > 0.05). This indicates its effectiveness in blocking the formation of peroxides. In addition, the extract showed an *F*2 parameter value between 0 and 1 (0.618 ± 0.10) and showed similar results to the quercetin control. These results corroborate the antioxidant potential of EEPC shown in this study.

The above results demonstrate the antioxidant capacity of *P. cauliflora* extract across a variety of oxidative pathways and provide strong evidence for EEPC as a significant antioxidant extract. As such, EEPC may be useful in preventing the development of chronic and non‐healing wounds, as these conditions are associated with redox imbalance and oxidative stress. The phenolic compounds in EEPC's composition are directly responsible for its antioxidant potential, mainly flavonoids and tannins [19, 39]. The presence of phenolic compounds increases the radical scavenging activity of EEPC. Therefore, the quantification of phenolic compounds can be directly correlated with the antioxidant content of the extract [40]. Also states that the antioxidant activity of polyphenols, such as those found in EEPC, is largely manifested through the scavenging of ROS [41].

### 2.5 Anti‐Inflammatory Properties

Nitric oxide (NO) is a mediator released by leukocytes and is strongly associated with the inflammatory phase of wound repair. However, a prolonged inflammatory environment and high NO production can result in deleterious effects on wound healing [42, 43]. Similar extract concentrations to those tested in the cell viability assay were chosen to assess NO production by peritoneal macrophages in vitro.

**FIGURE 4:**
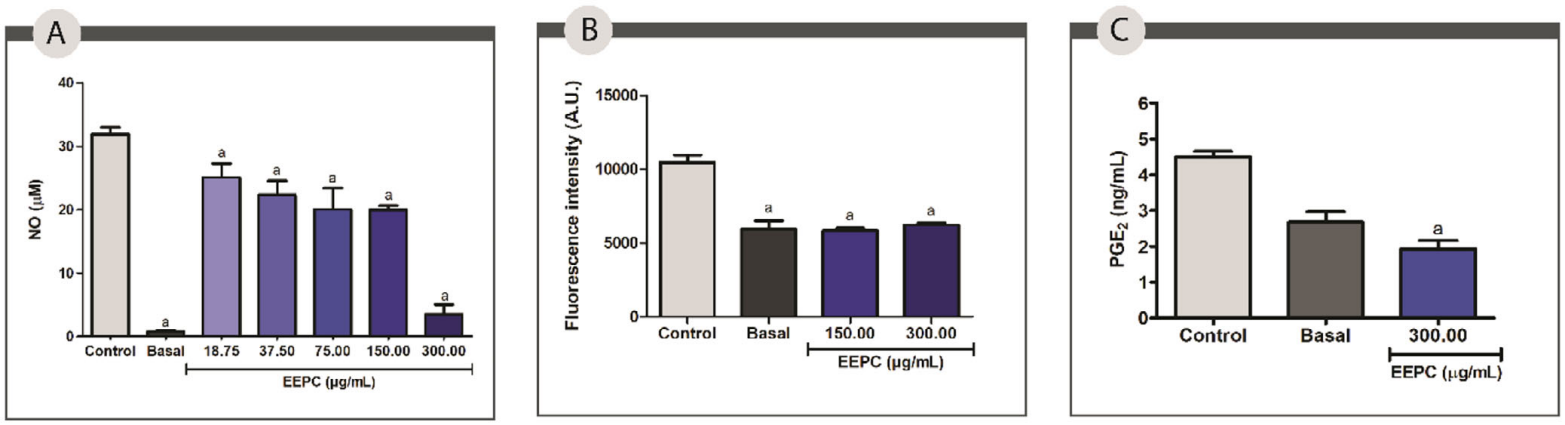
Anti‐inflammatory activity of the ethanolic extract from *Plinia cauliflora* leaves (EEPC). (A) Nitric oxide (NO); (B) Lipid droplets (LDs); and (C) Prostaglandin E2 (PGE_2_). Basal—Unstimulated cells treated with vehicle (DMSO). Control—Stimulated cells (IFN‐γ + LPS) treated with vehicle (DMSO). a—Statistical difference from control (*p* < 0.05). ANOVA followed by the Bonferroni test. The experiment was conducted in triplicate, and results are expressed as mean ± standard deviation (SD). Error bars represent SD.

EEPC inhibited approximately 21%, 24%, 36%, 64%, and 89% of NO production at 18.75, 37.50, 75.00, 150.00, and 300.00 µg/mL, respectively. The IC_50_ value was 76.12 ± 9.41 µg/mL. In addition to demonstrating the anti‐inflammatory potential of EEPC, the results presented in Figure 4A support the antioxidant activity of the extract, as NO is a reactive species. These findings are consistent with the high phenolic content described above, as these compounds are known to have potent anti‐inflammatory activity.

Leukocytes are among the cell types that produce lipid droplets (LDs), organelles that become more abundant in the cytoplasm once inflammation begins. It's been found that the production of prostaglandins and leukotrienes is related to the accumulation of LDs in leukocytes [44]. In this work, peritoneal macrophages of the BALB/c exposed to EEPC at 150.00 and 300.00 µg/mL were used to measure the intracellular levels of LDs (Figure 4B). Cells treated with the extract at 150.00 and 300.00 µg/mL had significantly reduced intracellular accumulation of LDs compared to the control (44% and 40%, respectively) (*p* < 0.05). The effect may be attributed to the ability of EEPC compounds to inhibit the synthesis of key inflammatory mediators, including prostaglandins [19].

To confirm this hypothesis, the ability of EEPC to reduce prostaglandin E2 (PGE_2_) production was also evaluated via enzyme‐linked immunosorbent assay (ELISA) in peritoneal macrophages of the BALB/c treated with EEPC at concentration of 300.00 µg/mL. The extract was able to reduce PGE_2_ production by approximately 52% was observed compared to the negative control (*p* < 0.05). This effect may be related to a reduction in the intracellular accumulation of LDs (Figure 4C).

The pro‐inflammatory interleukins TNF‐α, IL‐1β, IL‐6, and IL‐12, released by leukocytes, are linked to the recruitment of inflammatory cells to the injury site. Furthermore, IL‐6 is linked to enhanced fibroblast proliferation, and IL‐12 promotes the differentiation of naive T lymphocytes into effector lymphocytes [45]. Prolonged and excessive cytokine release promotes wound chronicity and ECM degradation by increasing the release of matrix metalloproteinases (MMP) [28, 29].

**FIGURE 5:**
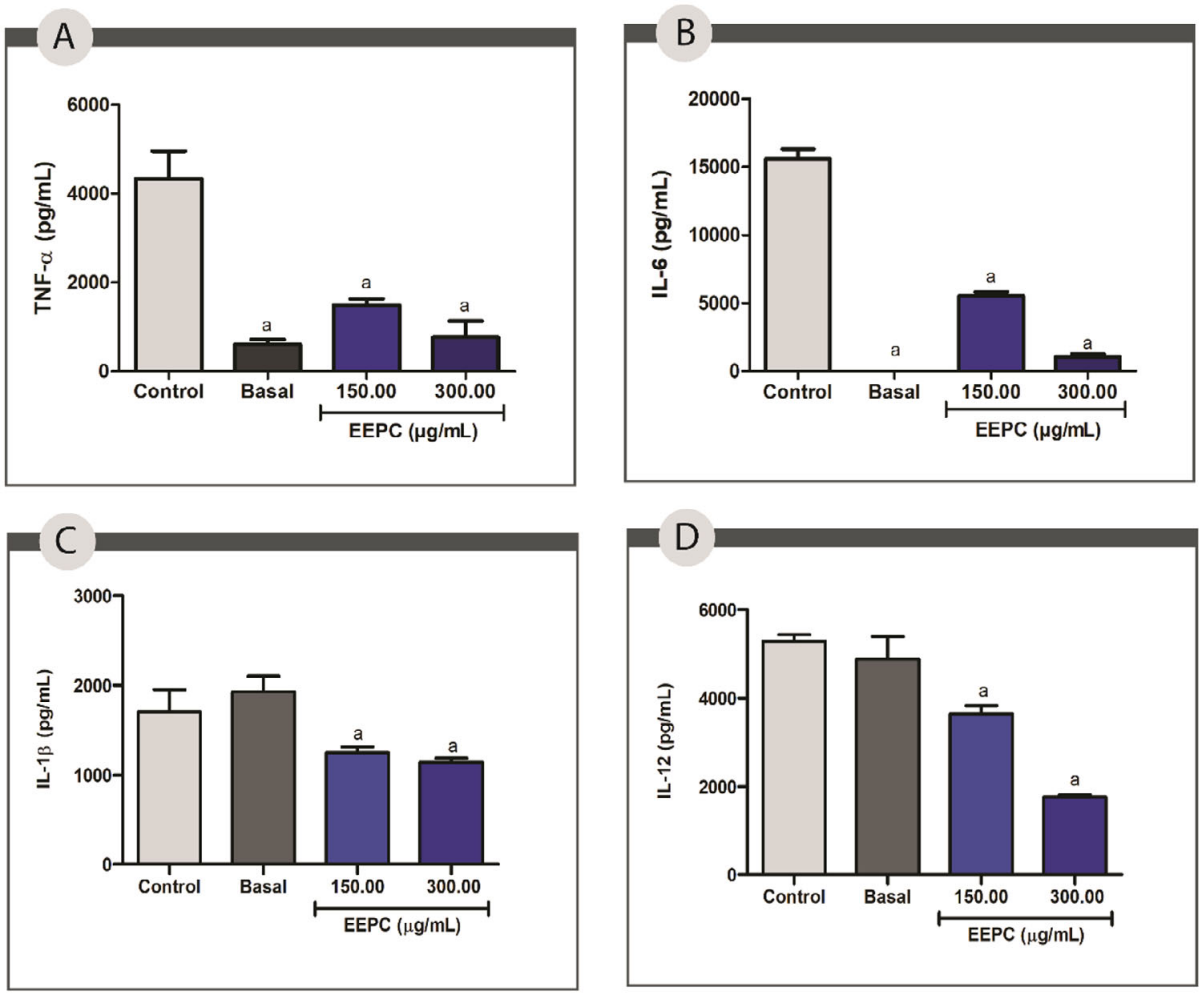
Cytokine production in the cells after treatment with the ethanolic extract from *Plinia cauliflora* leaves (EEPC). (A) TNF‐α; (B) IL‐6; (C) IL‐1β; and (D) IL‐12 expression by peritoneal macrophages of the BALB/c after treatment with EEPC. Control—Stimulated cells (IFN‐γ + LPS) treated with vehicle (DMSO). Basal—Unstimulated cells treated with vehicle (DMSO). a—Statistical difference from control (*p* < 0.05). ANOVA followed by the Bonferroni test. The experiment was conducted in triplicate, and results are expressed as mean ± standard deviation (SD). Error bars represent SD.

The production of TNF‐α, IL‐1β, IL‐6, and IL‐12 by cell cultures exposed to EEPC at 150.00 and 300.00 µg/mL was measured by ELISA. Compared to the control, EEPC decreased the levels of IL‐6 and TNF‐α by 65% and 50%, respectively, at 150.00 µg/mL, and by 96% and 74%, respectively, at 300 µg/mL (*p* < 0.05) (Figure 5A,B). The production of IL‐1 and IL‐12 was also significantly reduced by 40% and 64% at a concentration of 300 µg/mL and by 35% and 25% at 150 µg/mL (*p* < 0.05) (Figure 5C,D).

The anti‐inflammatory activity of EEPC may be associated with its rich composition of phenolic compounds and flavonoids. These compounds are known to have potent anti‐inflammatory properties, as they inhibit the production of pro‐inflammatory cytokines and enzymes [46]. Jaboticabin and ellagic acid have strong potential to inhibit pro‐inflammatory cytokines such as IL‐8 [47]. Quercetin, one of the flavonoids identified in EEPC, has extensive literature data supporting its anti‐inflammatory effect through the reduction of IL‐6 [48]. It is worth noting that, despite the significant effects observed at the highest extract concentration, the data are not indicative of cytotoxicity but rather of a highly significant modulating activity. In the cytotoxicity test, the samples demonstrated cell viability of over 70% at this concentration, indicating no interference in this regard. However, long‐term safety tests are required to determine the optimal concentrations for topical application.

The use of peritoneal macrophages from BALB/c mice in the present study is justified by their well‐documented immunological responsiveness and stability under experimental conditions. Notably, resident peritoneal macrophages exhibit a high degree of resistance to oxidative and cytotoxic stress, as demonstrated in recent human models. This characteristic makes them particularly suitable for evaluating the anti‐inflammatory property of test compounds, as it allows for a reliable assessment of cellular responses without confounding effects related to nonspecific cell damage. Additionally, BALB/c mice are widely used in immunopharmacological studies due to their consistent macrophage yield and well‐characterized immune profile, further supporting their use in mechanistic investigations involving inflammatory mediators and oxidative stress parameters [49].

### 2.6 Antimicrobial Activity

Microbial colonization of wounds can be one of the causes of complications. The healing of an infected wound is directly influenced by the presence of pro‐inflammatory mediators, which can even lead to the development of chronic wounds. Microbial colonization impairs patient recovery, can lead to complications, and has a financial impact [50]. *Pseudomonas aeruginosa, Escherichia coli, Staphylococcus aureus*, and *Candida albicans* are commonly found colonizing chronic wounds [51, 52, 53].

The antimicrobial potential of EEPC was first assessed by determining the minimum inhibitory concentration (MIC). According to the results (Table 3), the MIC of EEPC were 250 µg/mL against *P. aeruginosa*, 500 µg/mL against *S. aureus*, and 1000 µg/mL against *E. coli*, with a bactericidal effect against both. In addition, EEPC was fungicidal against *C. albican*s ATCC 24433 and ATCC 10231 with a MIC of 250 µg/mL.

**TABLE 3:** In vitro antibacterial and antifungal activity of ethanolic extract from *Plinia cauliflora* leaves (EEPC) for the microtiter dilution broth assay.

Microrganism	MIC with EEPCM (µg/mL)	Effect at MIC value	MBC or MFC (µg/mL)	MIC with azithromycin (µg/mL)	MIC with nystatin (µg/mL)
*Escherichia coli* (ATCC 10536)	1000.00	Bactericidal	1000.00	6.25	—
*Staphylococcus aureus* (ATCC 33591)	500.00	Bactericidal	500.00	400.00	—
*Pseudomonas aeruginosa* (INCAS 2742)	250.00	Bactericidal	250.00	100.00	—
*Candida albicans* (ATCC 24433)	250.00	Fungicide	250.00	—	2.50
*C. albicans* (ATCC 10231)	250.00	Fungicide	250.00	—	2.50

*Note*: Azithromycin is the positive control for bacterial strains; Nystatin is the positive control for fungal strains.

Abbreviations: MBC, minimum bactericidal concentration; MFC, minimum fungicidal concentration; MIC, minimum inhibitory concentration.

Azithromycin and nystatin were used as positive controls. Nystatin presented a MIC of 2.50 µg/mL against *C. albicans* species. Azithromycin, in turn, presented a MIC of 6.25 µg/mL against *E. coli*, 100 µg/mL against *P. aeruginosa*, and 400 µg/mL against *S. aureus*. Considering the values recommended by the CLSI, azithromycin should reach an MIC value of 8 µg/mL against *S. aureus* [54]. Therefore, it can be considered that the species was resistant to azithromycin, which is a coherent result, as the *S. aureus* ATCC 33591 strain is classified as MRSA (methicillin‐resistant *S. aureus*). Due to the limited penetration of macrolides through the membrane of Gram‐negative bacteria, the MIC for *P. aeruginosa* also indicates resistance to azithromycin [55].

Reported greater efficacy of the leaf extract against *Enterococcus faecalis, E. coli, S. aureus*, and *C. albicans* compared to the fruit extract [16]. Investigated the antifungal activity of the hydroalcoholic extract of the leaves against *C. albicans*, *Candida krusei, Candida tropicalis*, and *Candida parapsilosis* and found promising effects, especially against *C. krusei* [19]. Found the bark extract to be effective at various MICs against *E. coli, Bacillus subitilis, P. aeruginosa*, and *S. aureus* [56]. Although the results presented have high MIC values, considering a plant‐based raw material, they can be considered adequate. Furthermore, considering its use in topical formulations for the treatment of skin lesions, the amount of active ingredient is bearable.

Due to their clinical significance in the context of wound colonization and the resistance shown by *S. aureus* and *P. aeruginosa*, these bacteria were selected for the following tests.

**FIGURE 6:**
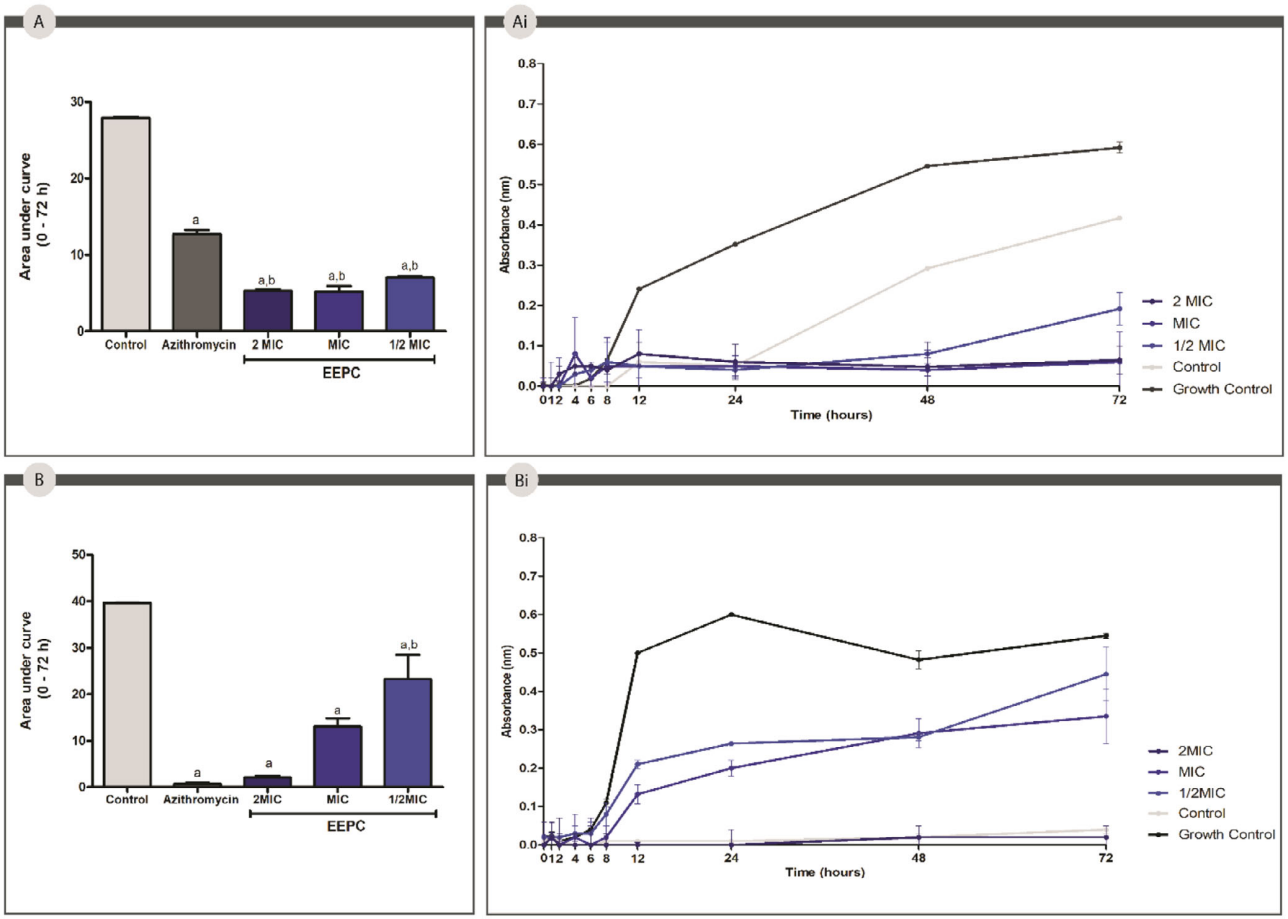
Growth kinetics of *Staphylococcus aureus* and *Pseudomonas aeruginosa* treated with the ethanolic extract from *Plinia cauliflora* leaves (EEPC), at concentrations of 2 MIC, MIC, and 1/2 MIC values along 72 h of incubation. Azithromycin, at the MIC value, was used as a positive control. (A) Area under the curve analyses (AUC) obtained from *S. aureus* growth kinetics. (Ai) *S. aureus* growth curve. (B) AUC analyses obtained from *P. aeruginosa* growth kinetics. (Bi) *P. aeruginosa* growth curve. a—Statistical difference from control (*p* < 0.05). b—Statistical difference from azithromycin (*p* < 0.05). ANOVA followed by the Bonferroni test. The experiment was conducted in triplicate, and results are expressed as mean ± standard deviation (SD). Error bars represent SD. MIC, minimum inhibitory concentration.

The evaluation of the bacterial growth kinetics is important to understand the antimicrobial activity of the respective extract over time. The area under the curve analyses (Figure 6) showed that EEPC and azithromycin were able to significantly (*p* < 0.05) inhibit the growth of *P. aeruginosa* over 72 h compared to the control group. About *S. aureus*, EEPC significantly (*p* < 0.05) reduced bacterial growth, with an even more effective reduction than the control, azithromycin. For *P. aeruginosa*, EEPC and azithromycin showed no statistically significant differences.

**FIGURE 7:**
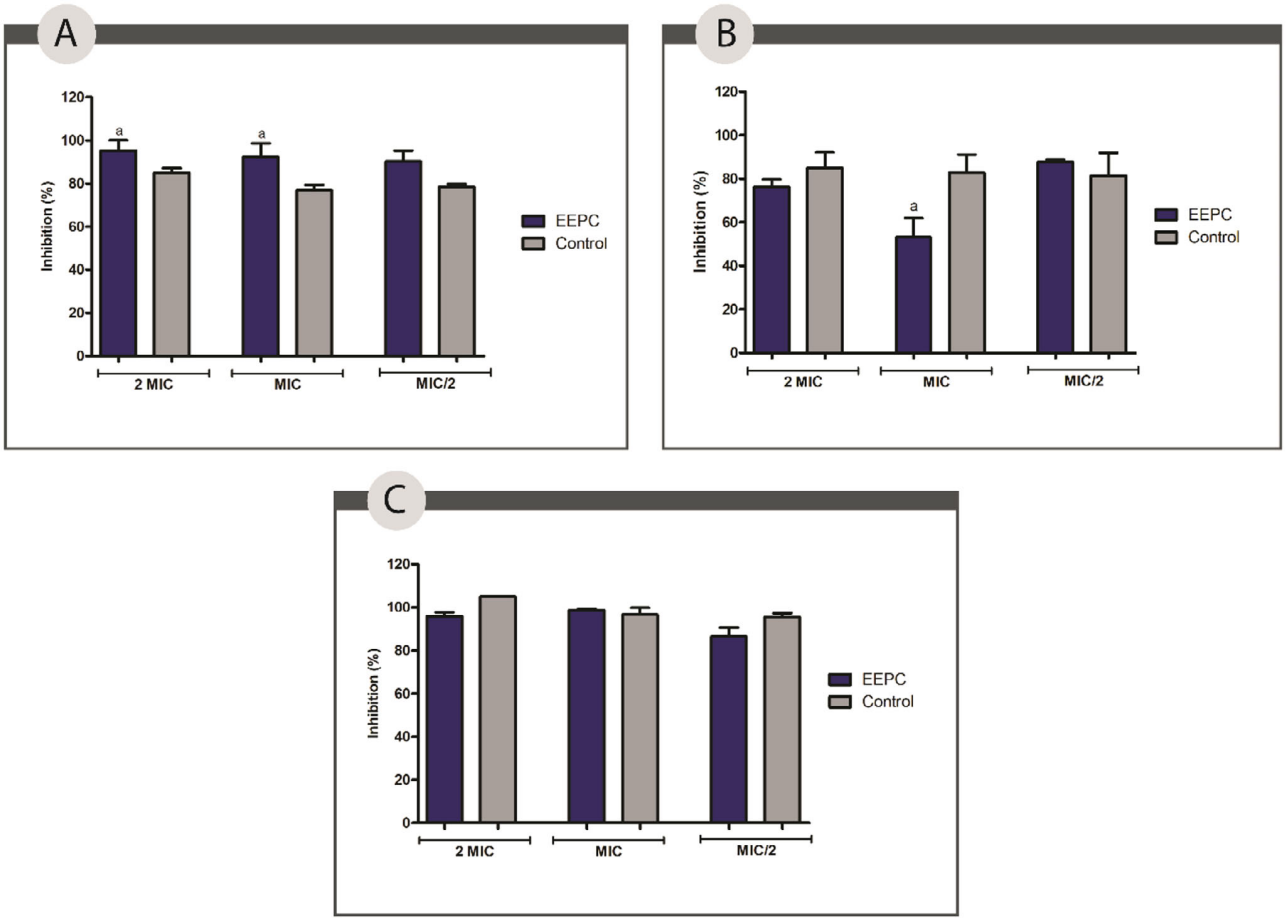
Percentage of inhibition of *Staphylococcus aureus* (A), *Pseudomonas aeruginosa* (B), and mixed (C) biofilms treated with the ethanolic extract from *Plinia cauliflora* leaves (EEPC), at the concentrations of 2 MIC, MIC, and 1/2 MIC values.

Regarding bacterial biofilm formation, EEPC was evaluated against *S. aureus* and *P. aeruginosa*. The results presented (Figure 7) show that EEPC at a concentration of 2 MIC inhibited 95.40%, a statistically significant difference (*p* < 0.05) compared to azithromycin against *S. aureus*. At MIC and 1/2 MIC, EEPC showed statistically similar inhibition percentages (*p* > 0.05) to the positive control, azithromycin. The same behavior was observed against *P. aeruginosa*, and the mixed biofilm EEPC at MIC, MIC, and 1/2 MIC concentrations showed a percentage of inhibition that was not statistically different compared to the positive control used.

Although not statistically significant in most of the tests, the result presented by EEPC at a concentration of 2 MIC against *S. aureus* is highly significant. One of the current challenges in wound care is the formation of MRSA biofilms, a mechanism of resistance and pathogenicity of the species. Due to its widespread resistance and ability to evade the immune system, MRSA biofilm on wounds is one of the major causes of complications and chronicity of lesions [57, 58].

Oliveira et al. assessed the effectiveness of a hydroethanolic extract derived from jabuticaba peel in inhibiting biofilm formation by *S. aureus* and *Acinetobacter baumannii* [51]. The authors found a significant reduction of 42.1% against *S. aureus* at a concentration of 11.1 mg/mL and 44.2% against *A. baumannii* at a concentration of 5.55 mg/mL. It is believed that the antimicrobial activity of the extract is mainly related to the presence of phenolic compounds such as flavonoids and tannins.

The combined action of these compounds works by disrupting the cell membrane, inhibiting enzymatic activity, and promoting the rupture of the bacterial biofilm [57, 58]. The antimicrobial results of the EEPC are groundbreaking. Other parts of the plant have already shown signs of antimicrobial efficacy, but no data were found specifically evaluating leaves in this respect.

### 2.7 Wound Healing Activity

Wound repair is a multi‐stage process, with specific cell types involved at each stage. During the proliferative phase, fibroblast migration and proliferation are of paramount importance for collagen production and cellular matrix formation to occur properly [12, 59]. Given the importance of fibroblasts at this stage, substances capable of increasing their migration and proliferation can directly increase wound repair [60].

**FIGURE 8:**
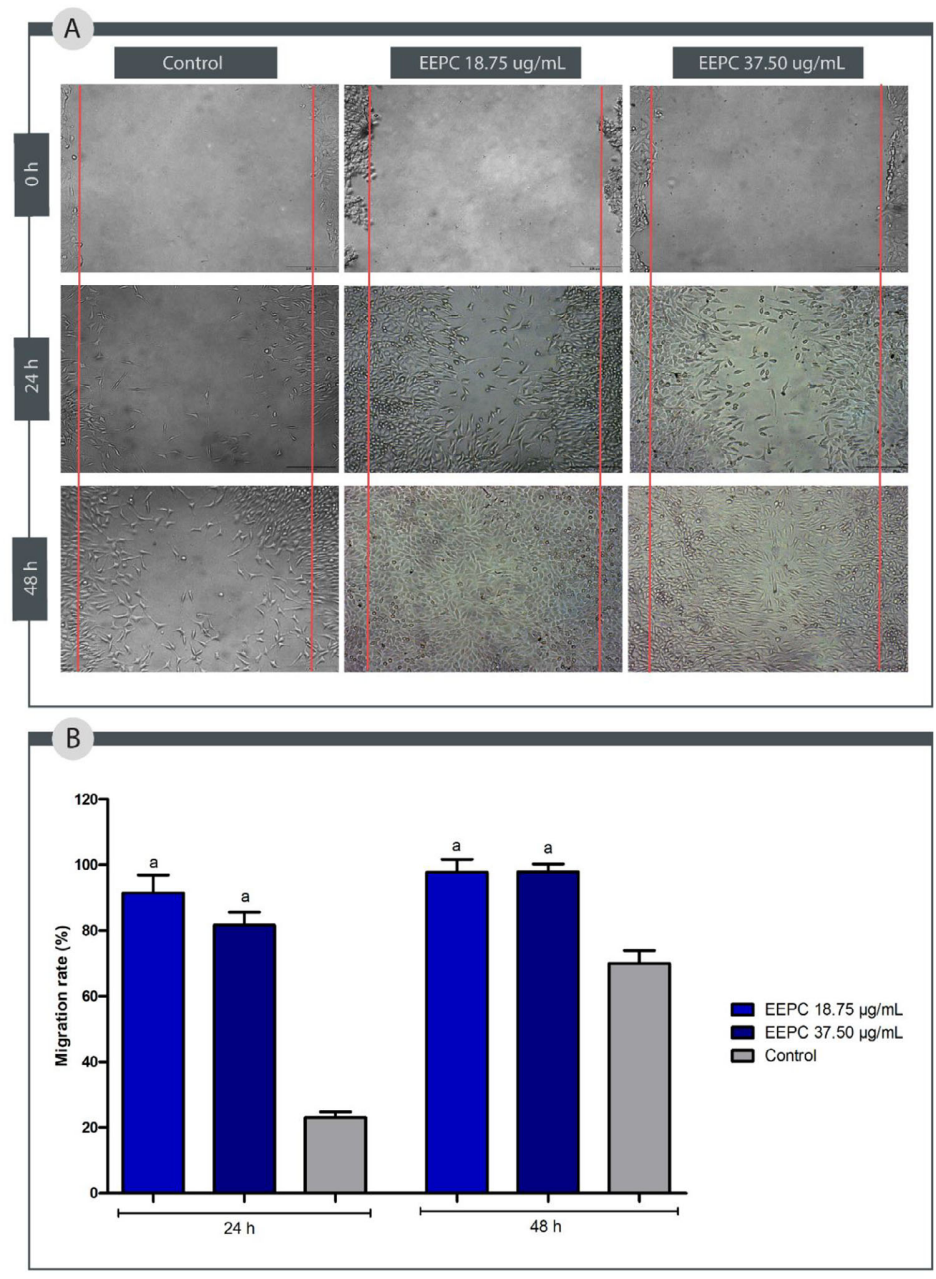
Effect of the ethanolic extract from *Plinia cauliflora* leaves (EEPC) on migration of fibroblasts L929. (A) Fibroblasts L299 treated with EEPC (18.75 and 37.50 µg/mL) and control group at times 0, 24, and 48 h after scratching. (B) Fibroblast L929 migration rate 24 and 48 h after scratching. a—Statistical difference from control (*p* < 0.05). ANOVA followed by the Bonferroni test. The experiment was conducted in triplicate, and results are expressed as mean ± standard deviation (SD). Error bars represent SD.

To simulate the process of fibroblast migration in the proliferative stage of a wound, the scratch test was performed. Compared to the control group, EEPC at both concentrations tested (18.75 and 37.50 µg/mL) accelerated the proliferation of fibroblasts L929 (Figure 8) (*p* < 0.05). After 24 h, EEPC at 18.75 µg/mL stimulated 91.45% of cell migration, whereas at 37.50 µg/mL stimulated 81.61%. After 48 h, EEPC at 18.75 µg/mL stimulated 97.66% of cell migration, whereas at 37.50 µg/mL stimulated 97.81%. These results indicate that EEPC is more effective in stimulating cell migration than the control group (24 h: 23.03% and 48 h: 69.97%).

Cefali et al. observed that an ethanolic extract from *P. cauliflora* fruit peels stimulated keratinocyte migration within 9 h [17]. In a separate investigation, a hydroalcoholic extract of *P. cauliflora* bark, tested at concentrations of 0.5, 5, 25, 50, and 100 µg/mL in a scratch wound healing assay, did not enhance the cell migration rate of L929 fibroblasts after 12 h; however, the authors noted that the extract did stimulate fibroblast proliferation [12].

According to the literature, the flavonoids and tannins present in this species can promote wound healing [61, 62]. Flavonoids, such as quercetin and rutin, have been reported to be effective in the treatment of diabetic wounds [61]. Tannins, such as tannic acid, also found in EEPC, have been used for many years to promote healing, but their mechanism of action is not yet well understood. This process is thought to be due to the strong antioxidant activity of these compounds and their ability to promote fibroblast proliferation [63, 64].

Interestingly, fibroblast (L929) proliferation was higher at lower concentrations of the extract, suggesting a biphasic response. This may result from the complex composition of the extract, where pro‐proliferative compounds (e.g., flavonoids) predominate at low doses, whereas cytotoxic or anti‐migratory components (e.g., tannins) prevail at higher concentrations [65, 66]. Further studies should investigate dose‐dependent effects on signaling pathways to optimize therapeutic use.

## 3 Conclusion

This study is the first to report the biological activity and phytochemical composition of the EEPC, emphasizing its potential application in the treatment of skin wounds. Thirteen compounds were identified, with gallic acid, epicatechin, quercetin, and rutin being the most prominent, and gallic acid as the major constituent. These phenolic compounds, particularly flavonoids and tannins, may serve as useful chemical markers for EEPC. The extract demonstrated a wide range of bioactivities, including antioxidant, anti‐inflammatory, antimicrobial, and wound healing properties. EEPC effectively inhibited lipid peroxidation and reduced intracellular ROS levels, indicating strong free radical scavenging activity. It also significantly suppressed the production of pro‐inflammatory mediators, including IL‐1β, IL‐6, IL‐12, TNF‐α, NO, LDs, and PGE_2_. Antimicrobial assays showed pronounced activity against *S. aureus* and *P. aeruginosa*, pathogens commonly associated with infected skin wounds, with a sustained reduction in bacterial growth observed over 72 h. Furthermore, EEPC significantly promoted the migration of L929 fibroblasts in vitro, supporting its potential role in enhancing wound repair. Importantly, this study underscores the therapeutic potential of an underexplored part of the *P. cauliflora* plant, its leaves, which constitute a significant portion of the plant's annual biomass. The valorization of this by‐product not only adds value to agricultural residues but also contributes to the reduction of agro‐industrial waste, supports local and regional economies, and fosters biodiversity conservation, aligning with key principles of sustainability and the United Nations Sustainable Development Goals. Nonetheless, the present study is limited by the absence of in vivo experimentation and comprehensive toxicity and safety assessments. Future research should focus on elucidating the underlying mechanisms of action, as well as establishing the pharmacological safety and efficacy of EEPC through rigorous preclinical and clinical investigations.

## 4 Experimental Section

### 4.1 Plant Material

*P. cauliflora* (DC.) Kausel (Myrtaceae) leaves were collected from the Federal University of Minas Gerais (UFMG) in Belo Horizonte, Brazil, coordinates 19°52′30.8″ S; 43°58′31.7″ W, in May 2022, SISGEN/Brasil A032F41. The specimen was identified as *P. cauliflora* (DC.) Kausel (Myrtaceae) by Professor Dr. Alexandre Salino, Curator of the BHCB Herbarium (UFMG), in 2014.

### 4.2 Extract Preparation

Approximately 2 kg of dried leaves were used to prepare the plant extract. The extraction process was carried out in stages, comprising five extraction cycles. PA‐grade ethanol was used as the solvent at room temperature for 15 days. Ethanol was selected due to its ability to extract phytochemicals of interest and its low toxicity, which helps preserve compounds sensitive to hydrolysis. The solvent was then removed using a BUCHI Labortechnik AG rotary evaporator (V‐700) under reduced pressure and at a water bath temperature of 45°C–55°C, resulting in the crude EEPC. The EEPC was stored in a glass container with a lid at 5°C.

### 4.3 Total Flavonoid Content

Using slight modifications, the total flavonoid content was assessed [67]. A stock solution of EEPC was prepared at a concentration of 1 mg/mL in ethanol. A UV–Vis spectrophotometer (Thermo Scientific Skanlt Multiskan GO, software 3.2) was used to measure the absorbance at 415 nm. Total flavonoid content was determined by taking the mean ± standard deviation (SD) in µg/mg of plant extract as rutin equivalents. The experiments were performed in triplicate.

### 4.4 Total Phenolic and Tannin Content

On the basis of the methods outlined, the total phenolic content (TPC) was estimated [68, 69]. A 1 mg/mL stock solution of EEPC was prepared using ethanol (EtOH). Tannic acid was used as the standard to construct the calibration curve. The absorbance was recorded at 770 nm using a UV–Vis spectrophotometer (Thermo Scientific Multiskan GO, software 3.2). The experiment was conducted in triplicates, and the TPC of the plant extract was expressed as µg/mg in TAE.

Tannin content was determined using the precipitation method, where gelatine was added to the samples. After centrifugation, the samples were filtered, and the filtered solution was analyzed for non‐adsorbed phenolics on the gelatin using the Folin–Ciocalteu method [68, 69]. The total tannin concentration in µg/mg of plant extract, expressed in TAE, was calculated by subtracting the non‐adsorbed phenolics from the total phenolics. The assay was performed in triplicate.

### 4.5 UPLC‐QTOF‐MS Analysis

UPLC‐QTOF‐MS (UPLC Shimadzu Nexera model, QTOF‐MS Bruker Compact model electrospray ionization source) was used for the chemical characterization of EEPC according to the outlined approach. The mobile phase used was acidified water, pH = 3, with formic acid (phase A) and methanol (phase B). The injection flow rate was set to 0.4 mL/min and the run time to 12 min. The column used was Kinetex 2.6 µm—C18—100A, length 100 mm × 3.0 mm. The chromatographic run started with 40% phase B at 0.01 min, reached 70% phase B at 8.20 min and 95% phase B at 9.70 min, with a subsequent return to 40% phase B at 10.20 min, the run ending at 12 min. Ionization conditions were in positive [M + H]^+^ mode with the following specifications: ion source electrospray voltage of 40 V, capillary voltage of 4500 V, and capillary temperature of 220°C. Full scan mass acquisition was performed by scanning from 100 to 1000 *m*/*z*.

### 4.6 HPLC‐DAD Analysis

The phenolic profile of EEPC was determined by HPLC‐DAD, in an Agilent Technologies 1200 Series, using a Zorbax SB‐C18 column (4.6 × 250 mm^2^, 5 µm) and DAD by the gradient method: 0–5 min 90% of water Milli‐*Q* (solvent A) with 1% orthophosphoric acid (pH ≈ 3.5) and 10% of acetonitrile (solvent B); 5–10 min 80% of A; 10–30 min 70% of A; 30–40 min 60% of A; 40–50 min 90% of A [70]. The flow rate was 0.5 mL/min for 50 min with an injection volume of 10 µL at an EEPC concentration of 1 mg/mL solubilized in acetonitrile, and the temperature was maintained at 25°C during the analysis. UV absorption spectra of standards and samples were measured between 200 and 400 nm. Gallic acid, epicatechin, rutin, and quercetin were identified by comparing their retention times and spectral characteristics with those of the standards, and their concentrations were determined using calibration curves. Each standard was dissolved in acetonitrile. The HPLC analysis was performed at 254 nm. Peak assignment was confirmed by injecting the standard mixture and co‐injecting EEPC and standards. The experiments were performed in triplicate. Calibration curves (ranging from 6.25 to 50.0 µg/mL) were constructed by plotting the average peak areas against the concentration of each standard. The amount of each compound was then determined using a regression equation.

### 4.7 Cell Viability

#### 4.7.1 Cell Culture Conditions

L929 fibroblast cells (ATCC CCL‐1 NCTC) were cultured in DMEM (Dulbecco's modified Eagle's medium), supplemented with 10% fetal bovine serum (FBS) and 1% antibiotics (penicillin and streptomycin). Peritoneal macrophages of the BALB/c were cultivated in RPMI‐1640 media supplemented with 2 mM l‐glutamine, 1% streptomycin, 5% FBS, and 100 U/mL penicillin. The cell lines were maintained at 37°C with 5% CO_2_ in a humidified incubator. Male BALB/c mice weighing 20–25 g at 30 days of age were obtained from the Centre for Reproductive Biology at the Federal University of Juiz de Fora. On May 10, 2018, the Ethics in Animal Experimentation Committee of the Federal University of Juiz de Fora approved the technique (Protocol Number: 07/2018‐CEUA). The choice of male animals aimed to avoid the potential variability introduced by hormonal fluctuations inherent to the estrous cycle in females, which are known to modulate macrophage function, cytokine production, and oxidative stress responses. Standardizing the sex of the animals helped ensure experimental consistency and reproducibility in the evaluation of the anti‐inflammatory effects of EEPC.

#### 4.7.2 MTT Reduction Test

Into 96‐well microplates, the macrophages and fibroblasts were seeded at a density of 5 × 10^3^ and 2 × 10^5^ cells/well, respectively, and exposed to EEPC in concentrations varying from 18.75 to 300.00 µg/mL. The negative control was 0.06% dimethyl sulfoxide (DMSO). Cytotoxicity was assessed using the 3‐(4,5‐dimethyl‐2‐thiazolyl)‐2,5‐diphenyl‐2H‐tetrazolium bromide (MTT) test, which measured cell viability after a 48‐h incubation period [71]. At 570 nm, absorbance was measured. The assay was performed three times, and the results were presented as the percentage of cell viability. Considering the standards established by ISO 10993‐5 [72], cell viability greater than 70% was considered acceptable for the cells.

### 4.8 Antioxidant Activity

#### 4.8.1 Evaluation of ROS Production

ROS levels in macrophages were assessed using 2′,7′‐dichlorodihydrofluorescein diacetate (H2DCFDA) [73]. As previously mentioned, cells were cultivated and incubated. LPS at 1 µg/mL and IFN‐γ at 1 ng/mL were used to excite the macrophages after they had been treated with EEPC (18.75–300.00 µg/mL). The basal group consisted of unstimulated cells, whereas the control group consisted of stimulated cells treated with DMSO (vehicle). After 48 h, the cells were treated with H2DCFDA (1 mM) for 30 min in the dark, and they were then rinsed in phosphate buffered saline (PBS). Culture supernatants were tested for ROS generation using fluorescence (FLx800, BioTek Instruments Inc., Winooski, VT, USA) at excitation and emission wavelengths of 485/528, respectively. The 50% inhibitory concentration (IC_50_) is presented in µg/mL, along with the mean ± SD of the fluorescence intensity (A.U) in the results. Three triplicates of each experiment were conducted.

#### 4.8.2 DPPH Scavenging Activity

The DPPH scavenging activity was assessed following the method, with some modifications [74]. The extract was dissolved into prepared as a stock solution at 1 mg/mL, which was then diluted in a 96‐well microplate to concentrations ranging from 0.49 to 250.00 µg/mL. After a 30 min incubation in the dark, absorbance was recorded at 517 nm using a UV–Vis spectrophotometer (Thermo Scientific Multiskan GO, software 3.2). Quercetin and rutin were used as positive controls. The results are presented in µg/mL as the mean ± SD of the inhibitory concentration (IC_50_) at 50%. Three triplicates of the experiment were conducted.

#### 4.8.3 Total Antioxidant Capacity

The total antioxidant capacity was measured using the phosphomolybdenum complex reduction test [32]. EEPC and the positive controls, quercetin and rutin, were dissolved in a hydroalcoholic solution to achieve a final concentration of 0.5 mg/mL. The absorbance of each sample was measured at 695 nm using a UV–Vis spectrophotometer (Thermo Scientific Skanlt Multiskan GO, software 3.2). The experiment was carried out in triplicate. The results were expressed as the mean ± SD of the relative antioxidant activity (RAA%) for quercetin and rutin, as shown below:

$$\mathrm{RAA}\%=\frac{\mathrm{Abs}\left(\mathrm{EEPC}\right)-\mathrm{Abs}\left(\mathrm{EEPC}\;\mathrm{blank}\right)}{\mathrm{Abs}\left(\mathrm{control}\right)-\mathrm{Abs}\left(\mathrm{control}\;\mathrm{blank}\right)}$$

where Abs (EEPC) represents the absorbance of the extract; Abs (EEPC blank) is the absorbance of the extract's blank; Abs (control) refers to the absorbance of the positive controls (quercetin and rutin); and Abs (control blank) denotes the absorbance of the positive controls’ blank.

### 4.9 Inhibition of Lipid Peroxidation

#### 4.9.1 MDA Assay

The inhibition of lipid peroxidation was measured using the MDA assay [75]. EEPC and the positive control (rutin) were dissolved at concentrations of 7.5, 15.00, and 20.00 mg/mL. After preparation, the samples, along with pulverized beef and distilled water, were stored at 5°C in sealed amber bottles for 7 days. In test tubes, 50 µL butylated hydroxytoluene (BHT), 1.25 mL thiobarbituric acid (TBA), 2.5 mL phosphoric acid at 1%, and 0.5 g of each sample were added. The absorbance of the supernatant containing the MDA–TBA complex was then measured at 535 nm using a UV–Vis spectrophotometer (Thermo Scientific Skanlt Multiskan GO, software 3.2). This procedure was repeated on 0, 2, 4, 6, and 8 days. A standard curve was constructed using the MDA standard, and the assay was conducted in triplicate.

#### 4.9.2 β‐Carotene/Linoleic Acid Assay

The antioxidant activity was measured at β‐carotene/linoleic acid assay, with slight adjustments [76]. A stock solution of the extract was obtained at 1 mg/mL and then diluted to concentrations ranging from 31.25 to 1000.00 µg/mL. The 96‐well microplate was incubated at 45°C, and absorbance was measured at 470 nm using a UV–Vis spectrophotometer (Thermo Scientific Multiskan GO, software 3.2) at the initial time (0) and at 15‐min intervals over a 2‐h period. Quercetin and rutin served as positive controls. The results were presented as the mean ± SD of the percentage of inhibition (%*I*), calculated from the absorbance reduction of the negative control (vehicle).

Using the tangent calculation, the oxidation curve of the extract, positive controls, and negative control were also assessed. *F1* values, representing or the ability to prevent the formation of peroxides, were determined between 15 and 45 min after the start of the reaction. *F2* values, indicating the capacity to prevent the formation of other radical species, were determined between 75 and 90 min after the start of the reaction. Experiments were performed in triplicate. %I, the *F1* and *F2* parameters were calculated using the following equations:

$$\%I=\frac{\mathrm{Abs}\;\mathrm{nc}-\mathrm{Abs}\;\mathrm{sample}}{\mathrm{Abs}\;\mathrm{nc}}\;\times \;100$$

$$Tg=\frac{\mathrm{Abs}\;\left(15^{\prime }\right)-\mathrm{Abs}\;\left(45^{\prime }\right)}{45-15}\;F1=\frac{Tg\;\mathrm{Abs}\;\mathrm{sample}}{Tg\;\mathrm{Abs}\;\mathrm{nc}}$$

$$Tg=\frac{\mathrm{Abs}\;\left(75^{\prime }\right)-\mathrm{Abs}\;\left(90^{\prime }\right)}{90-75}\;F2=\frac{Tg\;\mathrm{Abs}\;\mathrm{sample}}{Tg\;\mathrm{Abs}\;\mathrm{nc}}$$

where Abs (sample) refers to the absorbance of the extract and positive controls; Abs (nc) is the absorbance of the negative control; %*I* is the percentage of inhibition of lipoperoxidation; and *Tg* is the tangent.

### 4.10 Anti‐Inflammatory Activity

#### 4.10.1 NO Production

To evaluate NO generation indirectly, the Griess method, as described, was used to measure nitrite concentration [77]. After being exposed to EEPC at doses between 18.75 and 300.00 µg/mL, peritoneal macrophages of the BALB/c were incubated for one hour. Following stimulation with 1 ng/mL of IFN‐γ and 1 µg/mL of LPS, cells were incubated for 48 h as previously detailed in the evaluation of ROS production. A 96‐well microplate containing the supernatants and Griess reagent was incubated for 10 min at room temperature. A UV–Vis spectrophotometer (Thermo Scientific Skanlt Multiskan GO, software 3.2) was then used to detect the absorbance at 540 nm. The basal group consisted of unstimulated cells, whereas the control group consisted of stimulated cells treated with DMSO (vehicle). A sodium nitrite (NaNO_2_) standard was used to plot a standard curve. The findings are shown as the 50% inhibitory concentration (IC_50_) in µg/mL and the mean ± SD of NO (µM). Three triplicates of the experiment were conducted.

#### 4.10.2 Accumulation of LDs

The accumulation of LDs was calculated using a modified version [78]. EEPC was administered to peritoneal macrophages of the BALB/c at doses of 150.00 and 300.00 µg/mL. The cells were stimulated with LPS at 1 µg/mL and IFN‐γ at 1 ng/mL after 1 h of incubation, and they were then incubated for 48 h as previously mentioned in in the evaluation of ROS production. After washing with PBS, the cells were incubated for 20 min at 25°C with 200 µL of Nile Red (10 µg/mL). The fluorescence was then measured in triplicate using a spectrofluorometer (FLx800, BioTek Instruments Inc., Winooski, VT, USA), at an excitation wavelength of 485 nm and emission wavelength of 528 nm. The fluorescence intensity (A.U.) mean ± SD is used to represent the results.

#### 4.10.3 Cytokines and PGE_2_ Production

The sandwich ELISA was used to assess the synthesis of PGE_2_ and the pro‐inflammatory cytokines TNF‐α, IL‐1β, IL‐6, and IL‐12 in the culture supernatant. After being exposed to EEPC at doses of 150.00 and 300.00 µg/mL for cytokine dosage and 300.00 µg/mL for PGE_2_ dosage, peritoneal macrophages of the BALB/c were incubated for 6 h. Next, as previously mentioned in the evaluation of ROS production, the cells were stimulated with 1 µg/mL of LPS and 1 µg/mL of IFN‐γ. They were then incubated for 24 h. Cytokine concentrations in the culture supernatant were measured according to the manufacturer's instruction using a commercially available BD OptEIATM kit (BD Biosciences). Absorbance was measured at 450 nm using a UV–Vis spectrophotometer (Thermo Scientific Skanlt Multiskan GO, software 3.2). Cytokine and PGE_2_ concentrations were determined using a standard curve and are expressed in pg/mL and ng/mL, respectively. The experiment was performed in triplicate.

### 4.11 Antibacterial and Antifungal Activity

#### 4.11.1 Bacterial and Fungal Strains

The antibacterial activity of the extract was analyzed using the following strains: *E. coli* (ATCC 10536), *S. aureus* methicillin‐resistant (ATCC 33591), and *P. aeruginosa* (INCAS 2742). Prior to each experiment, the bacterial strains were cultivated in Mueller Hinton (MH) culture media for 24 h at 37°C. *C. albicans* (ATCC 10231), which is resistant to fluconazole, anidulafungin, itraconazole, and voriconazole, and *C. albicans* (ATCC 24433), which is susceptible to traditional antifungal treatment, were used in antifungal investigations. Before every experiment, the fungal strains were cultivated in Sabouraud (SB) culture media for 24 h at 35°C.

#### 4.11.2 Minimum Inhibitory Concentration (MIC)

The MIC was determined using the CLSI M07 methodology for bacteria and the CLSI M27 methodology for fungi [52]. In a 96‐well microplate, a stock solution of EEPC (2.5 mg/mL) was prepared with 20% DMSO and serially diluted to concentrations ranging from 1000 to 7.8 µg/mL. The blank (growth broth + extract) and growth control (growth broth + extract + inoculum) were also included in the experiment. Positive controls included azithromycin (400.00–3.12 µg/mL) for bacteria and nystatin (10.00–0.08 µg/mL) for fungi. The MIC was determined as the lowest dilution that showed complete inhibition of the tested strain. The experiment was conducted in triplicate.

#### 4.11.3 MBC and MFC

Aliquots of 10 µL were taken from the wells showing no visible growth and plated onto SD agar for fungi and MH agar for bacteria. The plates were incubated for 24 h at 35°C for fungi and 37°C for bacteria. The MBC and MFC were determined as the lowest concentration of the extract needed to kill the bacteria or fungi [79]. All experiments were conducted in triplicate.

#### 4.11.4 Bacterial Killing Assay

According to protocol was evaluated of the effect of EEPC on the growth curve of *P. aeruginosa* and *S. aureus* [79]. A 96‐well microplate was prepared with the extract at concentrations of 2 MIC, MIC, and 1/2 MIC, MH broth, and bacterial inoculum standardized to 0.5 McFarland (10^8^ CFU/mL). Absorbance at 595 nm was recorded at 0, 1, 2, 4, 6, 8, 12, 24, 48, and 72 h following incubation at 37°C. Bactericidal activity of EEPC was assessed by plotting absorbance values over time. Azithromycin at MIC levels was used as positive control, whereas the growth control consisted of bacterial strains in MH broth without the extract. Three triplicates of the experiment were conducted.

#### 4.11.5 Evaluation of EEPC on Biofilms Adhesion

The impact of EEPC on the growth curves of *P. aeruginosa* and *S. aureus* was evaluated using a modified version of the protocol [79]. Compared to the original protocol, a lower MIC value and another method for determining bacterial activity were used. In a 96‐well microplate, MH broth, a bacterial inoculum standardized to 0.5 McFarland (10^8^ CFU/mL), and the extract at concentrations of 2 MIC, MIC, and 1/2 MIC were combined. Absorbance at 595 nm was measured at 0, 1, 2, 4, 6, 8, 12, 24, 48, and 72 h after incubation at 37°C. Bactericidal activity of EEPC was determined by plotting absorbance values over time. Bacterial strains in MH broth without the extract served as growth control, whereas azithromycin at MIC levels was used as a positive control. The experiment was conducted in triplicate. The percentage of biofilm inhibition (%) was calculated using the following equation:

$$\mathrm{Biofilme}\;\mathrm{inhibition}\;\left(\%\right)=\frac{\mathrm{OD}\;\mathrm{control}-\mathrm{OD}\;\mathrm{treatment}}{\mathrm{OD}\;\mathrm{control}}\times \;100$$

Considering ODtreatment: optical density of the treatment sample; ODcontrol: optical density of the growth control.

#### 4.11.6 Scratch Wound Healing Assay

The impact of EEPC on the migration of L929 fibroblast was evaluated using a modified version of the protocol [80]. Briefly, 24‐well microplates were seeded with 5 × 10^4^ cells per well and allowed to adhere for 24 h. To create a linear scratch in the cell monolayer, a sterile 200 µL pipette tip was used. EEPC was applied to the scratched area at concentrations of 18.75 and 37.50 µg/mL, whereas control samples were treated only with fresh DMEM. Using the FLx800 microscope (BioTek Instruments Inc., Winooski, VT, USA), images were captured at 0, 24, and 48 h at 10× magnification. Cell migration was assessed by measuring the scratch width in each image, employing Olympus IX51 fluorescence microscopy software to track changes over time. The experiment was conducted in triplicate, and the cell migration rate was calculated using the following formula:

$$\begin{array}{ccc} \mathrm{Migration}\;\mathrm{rate}\;\left(\%\right) & = & \frac{\mathrm{Scratch}\;\mathrm{width}\;\left(t0\right)-\mathrm{Scratch}\;\mathrm{width}\;\left(tf\right)}{\mathrm{Scratch}\;\mathrm{width}\;\left(t0\right)}\\ & & \times \;100 \end{array}$$

where scratch width (*t*0) refers to the scratch width at time 0 h; scratch width (*tf*) refers to the scratch width at 24 or 48 h.

### 4.12 Statistical Analysis

Statistical analysis was performed using ANOVA followed by Bonferroni test (*p* < 0.05) with GraphPrism 8.0.1 software. Results are expressed as mean ± SD. All experiments were performed in triplicate (*n* = 3), with data representing at least three independent experiments.

## Author Contributions

Rodrigo Luiz Fabri, Leandro De Santis Ferreira, José Maria Barbosa‐Filho, Elaine Soares Coimbra, Luciana Moreira Chedier, and Gilson Costa Macedo acquired resources and conceived the tests. Priscila de Lima Paula and Mariana Hauck Vianna prepare the extract. Júlia Bertolini Fajardo, Priscila de Lima Paula, Ari Sérgio de Oliveira Lemos, Lara Melo Campos, Thalita de Freitas Souza, Thayná Gomes Ferreira, Maria Clara Machado Resende Guedes, Mariana Hauck Vianna, Ayrton Senna Pinheiro, Lívia Rodrigues Gamarano, and Ana Barbara Polo performed the experiments. Priscila de Lima Paula, Mariana Hauck Vianna, and Rodrigo Luiz Fabri analyzed the data. Rodrigo Luiz Fabri, Leandro De Santis Ferreira, José Maria Barbosa‐Filho, Elaine Soares Coimbra, and Gilson Costa Macedo provided with reagents and materials. Rodrigo Luiz Fabri, Lara Melo Campos, Júlia Bertolini Fajardo, Mariana Hauck Vianna, and Priscila de Lima Paula wrote and reviewed the article. The published version of the manuscript has been read and approved by all authors.

## Conflicts of Interest

The authors declare no conflicts of interest.

## Data Availability

The authors have nothing to report
